# The Role of Targeted Therapy in the Multi-Disciplinary Approach to Colorectal Liver Metastasis

**DOI:** 10.3390/cancers15133513

**Published:** 2023-07-06

**Authors:** Samantha M. Ruff, Alexander H. Shannon, Timothy M. Pawlik

**Affiliations:** Department of Surgery, Division of Surgical Oncology, The Ohio State University Wexner Medical Center and James Comprehensive Cancer Center, Columbus, OH 43210, USA; samantha.ruff@osumc.edu (S.M.R.); alexander.shannon@osumc.edu (A.H.S.)

**Keywords:** colorectal cancer, metastases, targeted therapy, liver

## Abstract

**Simple Summary:**

Colorectal cancer (CRC) is the second most common cause of cancer-related mortality in the United States. Despite best efforts, 5-year survival for unresectable metastatic CRC is only about 20%. CRC is a heterogeneous disease and the underlying genetic differences inform behavior, treatment strategy, and prognosis. Given the limitations of cytotoxic chemotherapy and the growing role of molecular profiling, research has focused on identifying and developing targeted therapies. We herein review how genetic profiling informs prognosis, crucial cell-signaling pathways that play a role in CRC carcinogenesis, and currently approved targeted therapies for metastatic CRC.

**Abstract:**

Colorectal cancer (CRC) is the second most common cause of cancer-related mortality in the United States. Among newly diagnosed patients with CRC, 20% will present with metastatic disease and another 25% will develop metastases. The surgical resection of the primary tumor and metastatic disease sites confers the best chance at long-term survival. Unfortunately, many patients will recur after resection or present with unresectable disease. As such, metastatic CRC is commonly treated with a combination of surgery, systemic therapy, and/or liver-directed therapies. Despite best efforts, 5-year survival for unresectable metastatic CRC is only about 20%. CRC is a heterogeneous disease and the underlying genetic differences inform behavior, treatment strategy, and prognosis. Given the limitations of cytotoxic chemotherapy and the growing role of molecular profiling, research has focused on identifying and developing targeted therapies. We herein review how genetic profiling informs prognosis, crucial cell-signaling pathways that play a role in CRC carcinogenesis, and currently approved targeted therapies for metastatic CRC.

## 1. Introduction

Colorectal cancer (CRC) is the second most common cause of cancer-related mortality for men and women in the United States [1]. Among newly diagnosed patients with CRC, 20% will present with metastatic disease and at least another 25% will develop metastases [2]. The lung and liver are both common sites of metastatic CRC (mCRC), but there is a propensity to metastasize to the liver due to the veinous drainage of the gastrointestinal tract into the portal system [3]. While the foundation of treating mCRC, surgical resection only treats macrometastatic disease. In turn, patients are vulnerable to recurrence from occult, disseminated micrometastatic disease. As such, the combination of surgery, systemic therapy (e.g., fluoropyrimidine-based chemotherapy, biologic therapy, immunotherapy), and/or regional therapy (e.g., hepatic artery infusion pump, Yttrium-90 radioembolization) is often employed to treat mCRC [2]. Despite best efforts, long-term survival is still poor for unresectable mCRC with 1-, 3-, and 5-year survival rates of approximately 70–75%, 30–35%, and 20%, respectively [2].

CRC is a heterogeneous disease and the underlying genetic differences between primary tumors can inform behavior, treatment strategy, and prognosis [4,5]. Furthermore, changes to the microenvironment play a crucial role in tumor development, progression, and response to treatment. Given the limitations of cytotoxic chemotherapy and the growing role that molecular profiling plays in guiding treatment for cancer, research has focused on identifying and developing potential targeted therapies for a more personalized approach. Targeted therapies disrupt the biologic features of cancer, like angiogenesis, thereby creating a balance between tumor cell death and off-target effects on healthy cells [6]. According to a recent meta-analysis, the use of chemotherapy with targeted therapy for unresectable mCRC was associated with an increase in overall response rate (68%) compared with chemotherapy alone (43%) [7]. We herein review how genetic profiling informs prognosis, crucial cell-signaling pathways that play a role in carcinogenesis, and currently approved targeted therapies for mCRC.

## 2. Genetic Profiling of mCRC

### 2.1. Consensus Molecular Subtypes

#### 2.1.1. CMS 1—Microsatellite Instability/Immune

**Figure 1:**
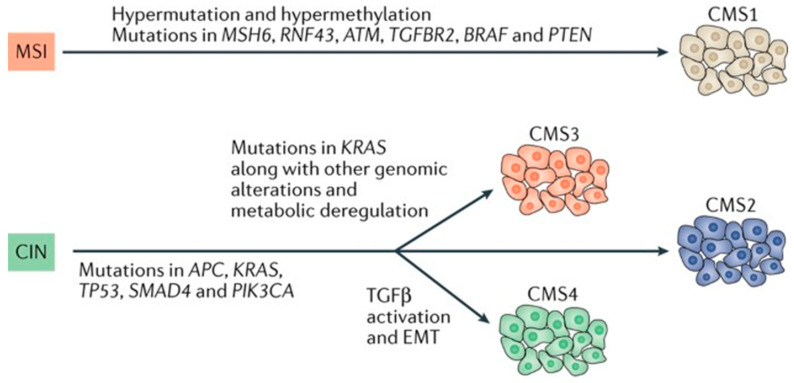
Colorectal carcinogenesis and transcriptomic subtypes. Abbreviations: consensus molecular subtypes (CMS), chromosomal instability (CIN), and microsatellite instability (MSI). This figure was reprinted with copyright permission from the study [15].

Guinney et al. established four robust consensus molecular subtypes (CMS) of CRC using gene expression data from 4151 patients [8]. Prior to this study, CRC was primarily defined by individual gene mutations (e.g., RAS or BRAF mutations) or microsatellite instability status. Guinney et al. created a new taxonomy of CRC to facilitate research and advance drug development strategy. Studies have demonstrated that these molecular and immune signatures can be used to predict clinical behavior and response to therapy [9]. CMS 1 tumors are hypermethylated, hypermutated, and have an immune-rich tumor microenvironment (Figure 1) [8]. The MLH1 promoter hypermethylation leads to defective mismatch repair (MMR) and microsatellite instability (MSI) [10]. In addition, nearly 80–90% of sporadic CMS 1 tumors carry a BRAF V600E mutation [8]. On histopathological assessment, CMS 1 CRC displays solid, trabecular, and/or mucinous features [10]. While early stage MSI tumors often carry a good prognosis, MSI and BRAF mutations in metastatic disease confer worse survival compared with other molecular subtypes [11,12,13]. These data are consistent with published data demonstrating that patients with CMS 1 metastatic disease have worse survival versus patients with CMS 2–4 metastatic disease [14].

#### 2.1.2. CMS 2—Canonical

While CMS 1 tumors are characterized by hypermethylation, CMS 2, CMS 3, and CMS 4 tumors arise from increased chromosomal instability. CMS 2 tumors follow the traditional CRC carcinogenesis pathway secondary to APC, p53, and RAS mutations (Figure 1) [8]. These tumors demonstrate the upregulation of the downstream targets of WNT and MYC and the increased expression of the oncogene epidermal growth factor receptor (EGFR), erythroblastic oncogene B-2 (ERBB2, also known as HER2), insulin-like growth factor 2 (IGF2), insulin receptor substrate 2 (IRS2), and transcription factor hepatocyte nuclear factor 4α (HNF4α) [16]. On pathologic assessment, these tumors display a complex tubular structure [10]. Compared with other subtypes, CMS 2 has the highest percentage of long-term survivors [8,17].

#### 2.1.3. CMS 3—Metabolic

At the gene expression level, CMS 3 was the most similar to normal colon tissue. This tumor type commonly demonstrates the enrichment of several metabolic pathways secondary to the KRAS activating mutations (Figure 1) [8]. On a pathologic assessment, these tumors display a papillary morphology [10]. Both CMS 2 and CMS 3 tumors are considered “cold” tumors with poor immune infiltration [18]. Given the high frequency of KRAS mutations, these tumors typically have a poor response to EGFR inhibitors [19].

#### 2.1.4. CMS 4—Mesenchymal

CMS 4 tumors demonstrate the upregulation of genes linked to epithelial–mesenchymal transition and signatures associated with activation of transforming growth factor-β (TGF-β), angiogenesis through the activation of vascular endothelial growth factor receptor (VEGFR), and matrix-remodeling pathways (Figure 1) [8]. On pathologic assessment, these tumors display a desmoplastic reaction with high stromal content [10]. Both CMS 1 and CMS 4 are considered “hot” tumors and are characterized by immune infiltration [18]. CMS 4 tumors have a higher rate of recurrence and worse overall survival (OS) than other CMS subtypes [8,14,20].

#### 2.1.5. CMS Subtypes and Tumor Location

Loree et al. compared mutation prevalence by anatomic site through the next generation sequencing of 1876 CRC tumors [21]. Right-sided tumors had higher rates of BRAF, PIK3CA, CTNNB1, SMAD4, and KRAS mutations, mucinous histology, and increased incidence of MSI. Left-sided tumors had higher rates of TP53 mutations. There were differences in the mutational profile based on anatomic location beyond the classic right, left, and rectal locations. For example, even though both are considered “right”-sided tumors, cecal carcinomas had a higher rate of RAS mutations (70%) and lower rate of BRAFV600 mutations (10%) compared with hepatic flexure carcinoma (RAS: 43%, *p* = 0.0001, BRAFV600: 22%, *p* < 0.0001). Differences in gene expression were also noted among “left”-sided tumors, as well as transverse versus right-sided tumors. The analysis of 608 tumors revealed a correlation between CMS and tumor site. Specifically, CMS 1 was associated with right-sided tumors, increasing in incidence from cecum (29%) to ascending colon (41%) to hepatic flexure (52%). Additionally, CMS 1 tumors were less common outside of the right colon. CMS 2 was most common in the sigmoid and rectosigmoid region [21].

### 2.2. Genetic Profiling Can Predict Recurrence after Resection for CRC Liver Metastases

After the resection of CRC liver metastases (CRC-LM), there is still a high risk of recurrence or development of new metastatic disease. The 5- and 10-year OS is predicted to be up to 50% and 25%, respectively [22,23,24]. The Fong clinical risk score evaluates risk based on clinical factors: node positive primary tumor, number of hepatic tumors, largest hepatic tumor size, disease free interval between primary and metastases, and carcinoembryonic antigen (CEA) level [25]. A Fong score of ≥3 is considered high risk for recurrence and confers a 20% 5-year survival after metastectomy. However, given the progress in recent years with the genomic sequencing of tumors, specific mutational profiles can also be utilized to predict long term outcomes. As discussed earlier, patients with CMS 1 who develop metastatic disease have poor prognosis compared with metastatic CMS 2/3/4 tumors. Additionally, patients with CMS 4 subtype have more aggressive disease with a higher recurrence rate and worse overall survival [8].

In a retrospective study, 98 patients who underwent repeat hepatectomy for mCRC-LM were evaluated for RAS mutations. After repeat hepatectomy, patients with RAS mutations (35% of population) had worse median recurrence free survival (RFS, 6.1 months) and median overall survival (OS, 26.6 months) versus patients with RAS wild-type tumors (RFS: 12.2 months, *p* = 0.03, OS: 42.5 months, *p* < 0.01) [26]. Furthermore, patients with RAS mutations are more likely to develop lung recurrences after resection for CRC-LM [27]. BRAF mutation also conferred worse OS and increased risk of recurrence among patients who undergo hepatic resection for CRC-LM [28,29].

## 3. Vascular Endothelial Growth Factor (VEGF)

**Figure 2:**
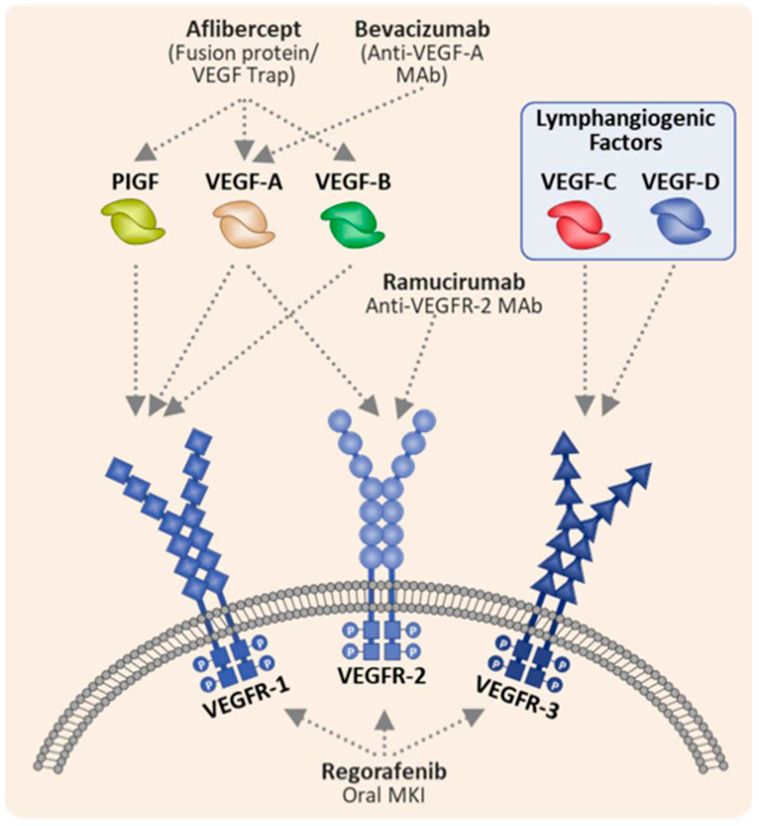
Mechanism of action of anti-VEGF agents in colorectal cancer. Abbreviations: placenta-derived growth factor (PIGF), vascular endothelial growth factor (VEGF), vascular endothelial growth factor receptor (VEGFR), multi-kinase inhibitor (MKI), and mAb (monoclonal antibody). This figure was reprinted with permission from an open access journal [38].

Angiogenesis is the process of creating new blood vessels to support cell growth, proliferation, organization, and survival. This process enables oncogenesis and metastases by facilitating the dissemination and survival of cancer cells. Cancer cells overexpress pro-angiogenic factors, like vascular endothelial growth factor (VEGF), platelet-derived growth factor (PDGF), and fibroblast growth factor (FGF), to establish an environment that promotes survival and proliferation. Newly formed blood vessels increase nutrient and oxygen delivery while simultaneously allowing for the removal of metabolic waste [30,31]. VEGF is a family of five proteins (VEGF-A, -B, -C, -D, and placental growth factor (PIGF)) that bind endothelial cells through three different tyrosine kinase VEGF receptors (VEGFR-1, -2, and -3). Angiogenesis is primarily mediated through the binding of VEGF-A, VEGF-B, and PIGF to VEGFR-1 and VEGFR-2, whereas lymphangiogenesis is mediated through interactions between VEGF-C and VEGF-D with VEGFR-3. These interactions play a significant role in cancer angiogenesis, progression, and migration through lymphatic channels (Figure 2) [32,33]. Studies have demonstrated in several cancers, including CRC, that increased levels of VEGF and VEGFR are associated with a worse prognosis [34,35,36,37].

### 3.1. Bevacizumab

Bevacizumab is a monoclonal antibody that selectively binds to circulating VEGF-A and inhibits its ability to bind to VEGFR. This process causes the regression of existing tumor vasculature, decreases the development of new vessels to prevent tumor growth, and normalizes tumor vasculature to improve chemotherapy delivery [39,40]. The AVF2107 trial randomized 813 patients with previously untreated mCRC to receive either bevacizumab and irinotecan/fluorouracil/leucovorin (IFL) or chemotherapy alone [41]. Compared with chemotherapy alone, the combination of bevacizumab/IFL led to improved median OS (20.3 months vs. 15.6 months), median PFS (10.6 months vs. 6.2 months), and a median duration of response (10.4 months vs. 7.1 months). Based on results of this trial, bevacizumab with fluorouracil-based chemotherapy was approved by the FDA as a first-line therapy for patients with mCRC [41]. It should be noted, however, that bevacizumab can lead to poor wound-healing and an increased risk of bleeding in the surgical setting [42,43].

The elderly patient population is historically underrepresented in clinical trials, given the propensity to enroll patients with lower ECOG scores. As such, it can be challenging to extrapolate clinical trial data to a more generalized population. Given the potential adverse effects of bevacizumab, like chronic heart failure, stroke, and myocardial infarction, there was some concern among oncologists about treating the elderly population with bevacizumab [44]. In response to these concerns, the phase III AVEX trial enrolled patients ≥70 years old with previously untreated mCRC who were not candidates for oxaliplatin- or irinotecan-based regimens to receive bevacizumab and capecitabine or capecitabine alone [45]. The data demonstrated that bevacizumab was safe and effective for this patient cohort.

Bevacizumab has also been studied among patients with progressive mCRC. In a multi-center phase III trial patients with mCRC who progressed on bevacizumab and first-line chemotherapy were randomized to receive second-line chemotherapy with or without bevacizumab [46]. This study demonstrated that bevacizumab plus second line chemotherapy resulted in a median OS of 11.2 months versus 9.8 months in the second line chemotherapy alone cohort. Even in the setting of refractory mCRC, continuing bevacizumab with second-line chemotherapy demonstrated clinical benefits. Additionally, Giantonio et al. randomized 829 patients with mCRC who progressed on fluoropyrimidine and irinotecan to receive FOLFOX4, FOLFOX4 with bevacizumab, or bevacizumab alone [47]. The authors reported that bevacizumab/FOLFOX4 cohort was associated with an improved median PFS and OS (PFS: 7.3 months, OS: 12.9 months) versus FOLFOX4 alone (PFS: 4.7 months, OS: 10.8 months) or bevacizumab alone (PFS: 2.7 months, OS: 10.2 months). The overall response rate (ORR) was 22.7%, 8.6%, and 3.3% for the FOLFOX4/bevacizumab, FOLFOX4 alone, and bevacizumab alone cohorts, respectively. Bevacizumab may normalize blood flow to the tumor and improve the delivery of chemotherapy. This synergistic effect, which was supported by the data in these trials, suggests that continuing bevacizumab with a different form of chemotherapy may be beneficial in refractory disease.

Bevacizumab has been demonstrated to be effective regardless of anatomic location (left vs. right primary tumor) and KRAS status (wild type vs. mutated) [45,48,49]. Several trials have also studied bevacizumab as adjuvant therapy after resection for stage II or stage III colon cancer, but none have reported a survival benefit, including NSABP-C08, AVANT, and QUASAR 2 trials [50,51,52].

### 3.2. Aflibercept

Aflibercept is a human recombinant fusion protein that acts as a decoy receptor for VEGF-A, VEGF-B, and PGF [53]. Aflibercept has a higher affinity for VEGF-A than bevacizumab or VEGFR. In addition, its ability to target PIGF in addition to VEGF may lead to increased efficacy over other anti-angiogenic agents [54]. Phase II trials in patients with mCRC failed to show the efficacy of aflibercept as a monotherapy [55]. Given the success of combination bevacizumab and chemotherapy as first-line therapy in mCRC, a phase II trial randomized 236 patients with mCRC to receive modified FOLFOX6 with or without aflibercept as first-line therapy (AFFIRM trial) [56]. There was no difference in PFS between the two treatment groups and the addition of aflibercept was associated with higher toxicity (increased hypertension, proteinuria, and deep vein thrombosis/pulmonary embolism).

The VALOUR phase III trial randomized patients with mCRC previously treated with oxaliplatin to receive FOLFIRI with either aflibercept or placebo [57]. About 30% of patients in the study population received previous bevacizumab therapy and this was balanced between the two arms. There was an improvement in median PFS (6.9 months vs. 4.7 months), median OS (13.5 months vs. 12.1 months), and response rate (19.8% vs. 11.1%) in the aflibercept cohort versus placebo, respectively. On the basis of this trial, the FDA-approved aflibercept for use in combination with FOLFIRI in patients with mCRC who progressed on oxaliplatin-containing regimens. Currently, there is an ongoing phase III trial (NCT03530267) evaluating aflibercept and 5-FU versus FOLFOX as first-line therapy in elderly or frail patients with mCRC.

### 3.3. Ramucirumab

Ramucirumab is a humanized monoclonal that binds to VEGFR-2 and blocks the binding of VEGF-A and VEGF-B. The phase III RAISE trial enrolled 1072 patients with mCRC who progressed on first-line therapy and randomized patients to receive FOLFIRI with ramucirumab vs. placebo [58]. The ramucirumab cohort had improved median PFS (5.7 vs. 4.5 months) and OS (13.3 months vs. 11.7 months) compared with the placebo cohort. Ramucirumab was approved by the FDA for patients with mCRC who progressed on FOLFOX and bevacizumab. Recently, phase III trials have demonstrated the efficacy of aflibercept, ramucirumab, or continuation of bevacizumab as second-line therapy in mCRC. These three regimens with second-line chemotherapy are all approved. Unfortunately, there are scarce data comparing these three anti-angiogenic agents directly. Bevacizumab has primarily been evaluated in comparison to FOLFOX or FOLFIRI, whereas aflibercept has only been evaluated in comparison to FOLFIRI [59]. High VEGF-D levels after progression on bevacizumab correlated with ramucirumab efficacy in the RAISE trial, which implies that with further investigation there may be a way to choose the optimal second-line therapy for patients through biopsies, genetic profiling, and/or tumor markers [58].

### 3.4. Tyrosine Kinase Inhibitors That Target VEGF/VEGFR

Ligands bind tyrosine kinase receptors (TKR) and activate downstream signaling pathways required for cell growth and proliferation. Tyrosine kinase inhibitors (TKI) bind TKR at the ATP-binding pocket and block other proteins from binding [60]. Regorafenib inhibits multiple TKR targets, including VEGFR. Regorafenib did not demonstrate efficacy as a first-line therapy but is currently approved as a second-line therapy for mCRC based on the CORRECT trial [61,62]. The CORRECT phase III trial randomized 760 patients with mCRC who progressed on standard therapy to receive either regorafenib or a placebo. Patients treated with regorafenib had improved median OS (6.4 vs. 5 months) and PFS (1.9 vs. 1.7 months) compared with the placebo [61,62]. Additionally, the CONCUR phase III trial evaluated the efficacy and safety of regorafenib in Asian patients with refractory mCRC [63]. This was the second trial to demonstrate that regorafenib confers a survival advantage. Furthermore, phase II trials have demonstrated that regorafenib is safe and effective in the elderly population [64,65]. So far, prospective trials combining regorafenib with chemotherapy have not demonstrated a survival advantage, but ongoing trials are still evaluating this potential regimen [66].

Fruquintinib is a highly selective TKI that blocks all three VEGFR. The encouraging results of phase I/II studies led to the phase III FRESCO trial [67,68]. This multicenter, placebo-controlled, double blind, randomized trial evaluated the use of fruquintinib in patients with mCRC who progressed on at least two lines of chemotherapy and had not been treated with a VEGF inhibitor. The median OS was 9.3 months with fruquintinib versus 6.6 months among patients who received placebo. Other TKIs with activity against VEGF/VEGFR are currently being evaluated for mCRC [66].

## 4. Epidermal Growth Factor Receptor (EGFR)

Erythroblastosis oncogene b (ERBB) is a family of four tyrosine kinase receptors with 11 growth factors. When ligands bind these receptors, downstream intracellular signaling pathways, including RAS/RAF/MEK/ERK, PI3K/AKT, and JAK/STAT3, are activated and lead to cell growth, survival, proliferation, metabolism, and migration. The four receptors include ERBB1 (EGFR/HER1), ERBB2 (Neu/HER2), ERBB3 (HER3), and ERBB4 (HER4) [69]. While the overexpression of EGFR has been noted in 25–77% of CRC, it is still unclear how EGFR impacts clinical outcomes [70]. Studies have noted an association between EGFR overexpression and poorly differentiated tumors. Additionally, in the pre-clinical setting, cell lines with EGFR overexpression demonstrate a propensity for increased cell proliferation and survival [71,72,73,74].

RAS is a proto-oncogene in the EGFR signaling pathway that activates the MAPK pathway and subsequent cell proliferation and survival. However, RAS mutations lead to the constitutive activation of the MAPK pathway and uncontrolled cell growth. Aggressive tumors, metastatic disease, and poor survival are also associated with RAS mutations [75,76,77,78]. Understanding the relationship between EGFR and RAS is critical given the clinical implications. In the presence of EGFR inhibitors, mutated RAS remains activated because it is downstream of EGFR [79]. Consequently, EGFR inhibitors should only be used in patients with RAS wild-type tumors.

### 4.1. Cetuximab

Cetuximab is a chimeric mouse–human monoclonal antibody that binds to the external domain of EGFR. The EGFR receptor is then internalized and degraded. Cetuximab is currently approved for the treatment of mCRC based on the results of the BOND trial [80]. This study randomized 329 patients with mCRC who had progressed on an irinotecan-based regimen to receive either cetuximab and irinotecan-based therapy or cetuximab monotherapy. Patients who received combination therapy had a longer median time to progression (4.5 months vs. 1.5 months, *p* < 0.001), compared with the cetuximab monotherapy cohort, but a similar median OS (8.6 months vs. 6.9 months, *p* = 0.48). The CRYSTAL phase III trial randomized 1198 patients with EGFR positive mCRC to receive either FOLFIRI and cetuximab or FOLFIRI alone as first-line therapy [81]. Patients in the FOLFIRI/cetuximab cohort had improved median PFS (8.9 months) compared to FOLFIRI alone (8 months), but there was no difference in OS. Cetuximab conferred a survival benefit versus best supportive care among patients with refractory mCRC and EGFR expression detectable on immunohistochemistry (IHC) [82]. The FIRE-3 phase III trial compared FOLFIRI/bevacizumab to FOLFIRI/cetuximab in patients with KRAS wild-type mCRC [49]. FOLFIRI/cetuximab resulted in a median OS of 28.7 months compared to 25 months in the FOLFIRI/bevacizumab cohort (*p* = 0.017).

Pre-clinical studies have suggested that treatment with VEGF inhibitors, like bevacizumab, may lead to resistance to anti-EGFR therapies, e.g., cetuximab [83]. Additionally, these early trials established that cetuximab is more effective in patients with KRAS wild-type mCRC. The recent TAILOR phase III trial was the first to evaluate the use of cetuximab/FOLFOX4 while prospectively choosing patients with RAS wild type mCRC [84]. Cetuximab/FOLFOX4 was associated with improved PFS and OS compared with FOLFOX4-only therapy.

### 4.2. Panitumumab

Panitumumab is a fully humanized monoclonal antibody that targets EGFR and has a lower risk of a hypersensitivity reaction compared with cetuximab [85]. The PRIME trial compared FOLFOX alone to FOLFOX/panitumumab in patients with KRAS wild-type mCRC [86]. The initial results demonstrated that the panitumumab cohort had a higher ORR and improved median PFS. The updated analysis confirmed an improvement in median OS for the panitumumab cohort. Additionally, this study further demonstrated that KRAS testing is crucial for patients prior to anti-EGFR therapy. The PARADIGM trial compared the use of FOLFIRI with panitumumab versus bevacizumab in patients with RAS wild-type mCRC [87]. Patients who received FOLFIRI/panitumumab had improved median OS (36.2 months vs. 31.3 months, *p* = 0.03) compared to FOLFIRI/bevacizumab, respectively.

Cetuximab and panitumumab are both approved for first-line treatment in KRAS wild-type mCRC. The ASPECCT trial compared panitumumab to cetuximab in patients with refractory mCRC but did not demonstrate a difference in survival [88]. Second line treatment for patients with KRAS wild-type mCRC should be guided by potential adverse events and toxicity of panitumumab and cetuximab. The National Comprehensive Cancer Network (NCCN) and European Society for Medical Oncology (ESMO) recommend either cetuximab or panitumumab in RAS and BRAF wild-type patients [89].

### 4.3. BRAF Mutation

BRAF encodes the BRAF protein kinase in the MAPK signaling cascade and its activation drives cell proliferation, differentiation, angiogenesis, and survival. Mutations in the BRAF gene are commonly due to a transversion mutation in exon 15 that results in a valine amino acid substitution (V600E). This mutation mimics regulatory phosphorylation and increases BRAF activity [90]. There are two subtypes: BM1, which is secondary to the activation of the KRAS/AKT pathway, and BM2, which is secondary to the dysregulation of the cell cycle and cell cycle checkpoints. This mutation is present in about 10% of patients with mCRC and is commonly found in women with MSI right-sided tumors with mucinous features [62]. Similar to patients with KRAS mutations, EGFR inhibitors are ineffective in BRAF mutated cancers.

While proven to be effective in other malignancies, like melanoma, BRAF inhibitors have not had the same success in mCRC. Vemurafenib has not been effective as a monotherapy for mCRC [91,92,93]. Pre-clinical studies suggested that this was due to the feedback activation of EGFR in the presence of BRAF inhibition [94]. Therefore, subsequent trials have shifted to evaluate combination BRAF and EGFR inhibitors. The BEACON trial randomized patients with refractory BRAF-mutated mCRC to receive either encorafenib (BRAF inhibitor), binimetinib (MEK inhibitor), and cetuximab (triplet therapy); encorafenib and cetuximab (doublet therapy); or cetuximab with FOLFIRI (control group) [95]. Median OS was 9 months, 8.4 months, and 5. 4 months for the triplet, doublet, and control cohorts, respectively. In addition to demonstrating the benefit of BRAF inhibitors in mCRC, this study was the first to demonstrate a survival benefit in mCRC patients with only targeted therapy and no chemotherapy. The American Society of Clinical Oncology (ASCO) guidelines recommend encorafenib and cetuximab as second-line therapy for patients with BRAF V600E-mutated mCRC [96].

### 4.4. Human Epidermal Growth Factor Receptor (HER2, Also Known as ERBB2)

HER2 is a transmembrane growth factor receptor that does not bind a specific ligand but rather is the preferred dimerization partner for other ERBB receptors. This process leads to the activation of downstream transcription pathways, including RAS/RAF/MAPK and PI3K/AKT, that promote cell proliferation and apoptosis [97]. HER2 overexpression is seen in 2–3% of patients with CRC and commonly detected in left-sided tumors [98,99]. Additionally, studies have described a discordance of approximately 14% between HER2 overexpression in the primary and metastatic tumor from the same patient. This suggests that even if a patient is HER2-negative in their primary tumor, the metastatic tumors should undergo testing for potential therapies [97].

While clinical trials evaluating the combination of a HER2-inhibitor and chemotherapy were terminated early due to low efficacy, studies with dual HER2-targeted therapies have shown more promise [100,101]. The MyPathway phase II trial treated 57 patients with HER2-amplified mCRC with trastuzumab and pertuzumab [102]. Median OS was 11.5 months and ORR was 32%. The TRIUMPH trial enrolled patients with RAS wild-type and HER2-positive mCRC with the same combination of trastuzumab and pertuzumab, and ORR was 30% [103]. An ongoing randomized trial is comparing trastuzumab and pertuzumab with cetuximab and irinotecan among patients with RAS wild-type, HER2-positive mCRC who progressed on initial therapy (NCT03365882).

Trials have also examined the combination of HER2-targeted therapy with a TKI. The HERACLES phase II trial treated patients with HER2-positive mCRC with combination trastuzumab and lapatinib (TKI-targeting EGFR and HER2) [104]. It is of note that the ORR was 28%, median PFS was 4.7 months, and median OS was 10 months. Increased ORR and PFS was associated with a higher HER2 gene copy. Additionally, this combination was effective in patients with previous resistance to the trastuzumab/pertuzumab regimen. The MOUNTAINEER study evaluated the use of trastuzumab with tucatinib (TKI for HER2) in patients with previously treated RAS wild-type, HER2-positive mCRC [105]. Median PFS was 8.1 months and median OS was 18.7 months. An ongoing study is continuing to evaluate this combination in an expanded cohort (NCT03043313).

### 4.5. KRAS Targeted Therapy

While not yet approved for mCRC, KRAS-targeted therapy has demonstrated effectiveness in other cancers [79]. Combining a KRAS inhibitor and an EGFR inhibitor may be a path to overcoming anti-EGFR therapy resistance in KRAS mutated patients. However, the challenge lies in ensuring only mutant KRAS is targeted to avoid the potential toxicity of inhibiting wildtype KRAS, which is required for normal cell function. Sotorasib and Adagrasib are KRAS selective inhibitors that bind to G12C KRAS mutants. Both were recently approved by the FDA for use in non-small cell lung cancer [106,107]. These agents are currently being tested in clinical trials with patients with mCRC and KRAS-G12C mutations (NCT04793958, NCT05198934).

## 5. Mismatch Repair Mutations (Microsatellite Instability)

**Figure 3:**
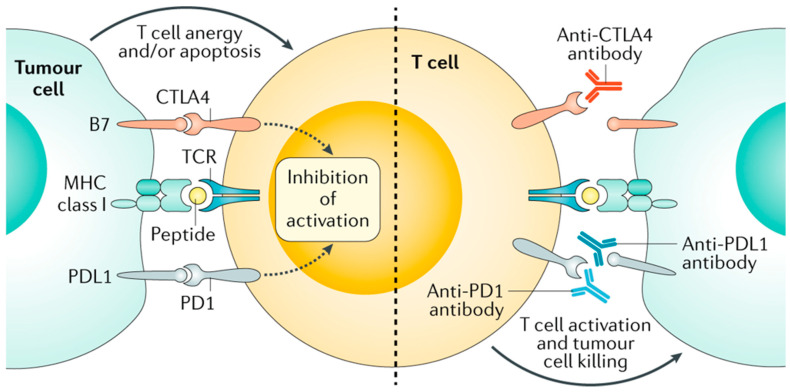
Endogenous peptides are processed and presented on major histocompatibility complex (MHC) class I molecules on the surface of all human cells, including cancer cells. The peptide–MHC complex is recognized by T cell receptors (TCRs). The response of the T cell is fine-tuned by a range of co-inhibitory or co-stimulatory signals. The ligands CD80 and CD86 of the B7 family of membrane-bound ligands can bind to the co-stimulatory CD28 and, especially in activated T cells, to cytotoxic T lymphocyte antigen 4 (CTLA4). Similarly, membrane-bound programmed cell death 1 ligand 1 (PDL1) and programmed cell death 1 ligand 2 (PDL2) can engage programmed cell death 1 (PD1), leading to T cell anergy and/or apoptosis. Monoclonal antibodies that bind to either the inhibitory receptors on T cells or their ligands on cancer cells antagonize inhibitory signaling and enable T cell activation and cytotoxic tumor cell killing. This figure was reprinted with copyright permission from [114].

Immune checkpoints are proteins that bind immune cell receptors and either inhibit or stimulate the immune system (Figure 3). Cancer cells evade the immune system by expressing inhibitory proteins and downregulating stimulatory proteins. Immune checkpoint inhibitors (ICIs) work to bolster the body’s natural immune response against cancer cells [108,109,110]. Patients with deficient mismatch repair (dMMR) and subsequent microsatellite instability (MSI) have a more favorable response to immune checkpoint inhibitors (ICI) [111,112]. These tumors have a high tumor mutational burden (TMB) and neoantigen load. Neoantigens are newly formed antigens from tumor mutations that can elicit a strong anti-tumor immune response [113]. dMMR is present in about 4% of patients with mCRC [96].

### 5.1. Pembrolizumab

In response to pro-inflammatory cytokines, programmed death ligand 1 (PD-L1) is expressed on somatic cells and binds to the programmed death 1 (PD-1) receptor to T cells, B cells, natural killer cells, dendritic cells, and myeloid-derived suppressor cells. When bound, there is the suppression of T cell migration, proliferation, and cytotoxin secretion [115]. Pembrolizumab is a PD-1 inhibitor and the first ICI demonstrated to be effective against dMMR mCRC. After several successful phase I trials, the phase II KEYNOTE-177 trial compared pembrolizumab to chemotherapy with or without bevacizumab as a first-line treatment in patients with MSI mCRC [116]. Pembrolizumab conferred superior median PFS (16.5 months) compared with chemotherapy/bevacizumab (8.2 months). Pembrolizumab is approved for dMMR/MSI high mCRC as first-line therapy.

### 5.2. Nivolumab

Nivolumab is also a PD-1 inhibitor and was approved in 2017 for MSI high or dMMR mCRC. The Checkmate-142 trial treated 74 patients with refractory MSI mCRC with nivolumab [117]. At a median follow-up of 12 months, the ORR was 31.1% and OS rate was 73.4%. A separate component of the Checkmate-142 trial evaluated the use of nivolumab and ipilimumab (CTLA-4 inhibitor) in patients with treatment-naïve MSI mCRC [118]. At a median follow-up of 29 months, the ORR was 69%. The median OS was not reached, but the 24-month OS rate was 79%. These data suggest that combination ICI may be more effective for patients with MSI mCRC.

## 6. Hepatocyte Growth Factor (HGF)/Mesenchymal-Epithelial Transition Factor (MET) Pathway

HGF is a ligand that binds the surface receptor MET to initiate various downstream pathways that help regulate hematopoiesis, organ regeneration, and wound-healing [69]. The overexpression of both MET and HGF is associated with poor prognosis in patients with CRC [69,119]. Furthermore, the overexpression of MET and EGFR is commonly noted in the same tumor. As such, MET and EGFR may affect each other and, in the presence of targeted therapy, give rise to compensatory activity from the other receptor. MET has been identified as a potential cause of EGFR inhibitor resistance [69].

### 6.1. Rilotumumab

Rilotumumab is a monoclonal antibody against HGF, which has mainly been studied in gastric and gastroesophageal cancers. Phase III studies examining rilotumumab (RILOMET-1 and RILOMET-2) were halted due to an increase in disease-related deaths [120,121,122]. In a randomized phase I/II trial of patients with KRAS wild-type mCRC, combination panitumumab with rilotumumab, ganitumab (anti-insulin-like growth factor 1 receptor), or placebo were assessed. Combination panitumumab and rilotumumab had an ORR of 31% versus 22% for patient treated with panitumumab/ganitumab [123].

### 6.2. Onartuzumab

Onartuzumab is a monoclonal antibody against the MET receptor, which has been evaluated in several malignancies. Unfortunately, onartuzumab has failed to demonstrate efficacy in phase III trials [124,125,126]. There was no differences in outcomes among patients with mCRC who were treated with FOLFOX/onartuzumab or FOLFOX/placebo. Sub-analysis of patients who were MET-positive on IHC staining similarly failed to demonstrate an effect for onartuzumab. As such, MET IHC was not a predictive biomarker [125].

## 7. Conclusions

Metastatic CRC requires a multidisciplinary approach involving the potential combination of surgery, cytotoxic chemotherapy, liver-directed therapy, and now targeted therapy. The underlying differences in the molecular profile of tumors and the microenvironments inform tumor biology, response to treatment, and prognosis. Targeted therapy allows for a more personalized approach by tailoring treatment to the unique features of the tumor. We are still in the early stages of understanding how to appropriately select patients who will best respond to individual therapies, but continued translational work is crucial to moving the field forward. In the pre-clinical setting, understanding the mechanisms of carcinogenesis, identifying novel targets, and developing new drugs will be important. Through large, multicenter clinical trials, tissues for sequencing should be obtained to elucidate the subtypes of mCRC and better identify the unique tumor characteristics that are associated with response to targeted therapy.
